# Incidence of Huntington disease in a northeastern Spanish region: a 13-year retrospective study at tertiary care centre

**DOI:** 10.1186/s12881-020-01174-z

**Published:** 2020-11-23

**Authors:** Paula Sienes Bailo, Raquel Lahoz, Juan Pelegrín Sánchez Marín, Silvia Izquierdo Álvarez

**Affiliations:** Departamento de Genética. Servicio de Bioquímica Clínica, Hospital Universitario Miguel Servet. C/ Padre Arrupe, s/n. Consultas Externas. Planta 3ª. 50009, Zaragoza, Spain

**Keywords:** Huntington disease, *HTT* gene, Incomplete penetrance alleles, Intermediate alleles

## Abstract

**Background:**

Despite the progress in the knowledge of Huntington disease (HD) in recent years, the epidemiology continues uncertain, so the study of incidence becomes relevant. This is important since various factors (type of population, diagnostic criteria, disease-modifying factors, etc.) make these data highly variable. Therefore, the genetic diagnosis of these patients is important, since it unequivocally allows the detection of new cases.

**Methods:**

Descriptive retrospective study with 179 individuals. Incidence of HD was calculated from the ratio of number of symptomatic cases newly diagnosed per 100,000 inhabitants per year during the period 2007–2019 in Aragon (Spain).

**Results:**

50 (27.9%) incident cases of HD (CAG repeat length ≥ 36) were identified from a total of 179 persons studied. The remaining 129/179 (72.1%) were HD negative (CAG repeat length < 36). 29 (58.0%) females and 21 (42.0%) males were confirmed as HD cases. The overall incidence was 0.648 per 100,000 patient-years. 11/50 positive HD cases (22.0%) were identified by performing a predictive test, without clinical symptoms. The minimum number of CAG repeats found was 9 and the most common CAG length among HD negative individuals was 16.

**Conclusions:**

Our incidence lied within the range reported for other Caucasian populations. Implementation of new techniques has allowed to determine the exact number of CAG repeats, which is especially important in patients with triplet expansions in an HD intermediate and/or incomplete penetrance allele, both in diagnostic, predictive and prenatal tests.

**Supplementary Information:**

The online version contains supplementary material available at 10.1186/s12881-020-01174-z.

## Background

Huntington’s disease (HD) is a progressive neurodegenerative disorder caused by the anomalous expansion of polyglutamine repeats (encoded by the trinucleotide CAG, Cytosine-Adenine-Guanine) in the first exon of the huntingtin gene (*HTT*, also known as *HD* or *IT15* gene), located in the short arm of chromosome 4 (4p16.3) (MIM#143100) [1].

HD is an autosomal dominant disease, so the risk that each child of an affected individual inherits the mutation is 50%. On the other hand, earlier age of onset in the offspring will depend on the size of the CAG_n_ fragment that it inherits, which tends to increase due to the phenomenon of clinical anticipation [2].

There is an inverse relationship between the number of CAG repeats and the age of onset of symptoms [3, 4]. In addition, the number of CAG repeats is also related to the age of death [5] and the rate of disease progression [6]. The repeated CAG trinucleotide in the general population is highly polymorphic, with sizes varying between 6 and 26 repeats. However, the diagnostic confirmation of HD involves identifying the presence of mutated alleles of the *HTT* gene with more than 36 repeats [7]. Alleles with 27–35 CAGs are called intermediate alleles (IAs) and are genetically unstable, being able to be amplified to pathological sizes in following generations through the paternal line, and exceptionally, through the maternal [8–11]. Finally, alleles with 36–39 repeats are related to an incomplete penetrance that is often associated with a later onset age and a slower disease progression rate. Those with 40 or more repeats show complete penetrance and inevitably lead to the clinical manifestation of HD [12, 13].

Some cases of HD occur sporadically in the population, making very difficult the precise estimate of the new mutation rate for a late onset disorder such as HD. It has been determined that 3% of affected patients may be the result of an expansion of the CAG triplet of an IA [14]. Thus, it is believed that the existence of these alleles acts as a reservoir from which de novo mutations arise in several generations [15].

In this way, the genetic study can be requested for confirmation of the clinical diagnosis of suspicion or as a presymptomatic or predictive diagnosis. There is also the possibility of prenatal diagnosis when one of the parents is a carrier of a pathological or unstable expansion, or even select healthy embryos in vitro for implantation (preimplantation genetic diagnosis) [2, 16].

The main objective of this study was to calculate the incidence of HD in Aragon population (Spain), who have been referred to the Clinical Genetics Section of the Hospital Universitario Miguel Servet (HUMS), reference centre in Aragon for the genetic study of HD, during the period 2007–2019, according to sex, clinical symptomatology and age at referral. Other objectives were to estimate the new mutation rate, to classify the patients diagnosed according to the CAG triplet repeats and to identify the allele in the polymorphic normal range that was most common. The number of cases per family were analysed to assess the paternal or maternal transmission of HD.

## Methods

### Subjects/patients

It was a descriptive retrospective study in which the study group comprised a total of 179 individuals from Aragon, who have been referred during the period January 2007–December 2019 to the Clinical Genetics Section of the HUMS, Zaragoza, Spain, for diagnostic or predictive testing for HD. Aragon is an autonomous community located in north-eastern Spain with a population of around 1.3 million with over half of it living in its capital city, Zaragoza. The area of Aragon represents a 9.43% of the surface of Spain, being thus the fourth autonomous community in size.

To carry out this study requires the presence of neurological and/or psychiatric symptoms compatible with the disease, or a family history information on HD that justifies it. A family history was considered positive when at least one other family member has clinical or genetic diagnosis of HD. A family history was considered as negative when no other siblings had signs or symptoms of HD, and the parents were both alive and healthy or did not have a neurological/psychiatric disorder. Demographical data including age at referral, sex, place of residence and family history are also recorded.

### Samples type

The type of primary sample used was peripheral blood with EDTA from which DNA extraction was performed, suitable sample for diagnostic studies and presymptomatic/predictive test. In prenatal studies, primary samples of chorionic villi and amniotic fluid were used.

### Methods/techniques

During the period 2007–2012 the determination of the CAG repeats in these samples was performed using a conventional polymerase chain reaction (PCR) followed by agarose gel electrophoresis being its main limitation the inaccuracy of not being able to quantify exactly the number of repeats of the triplet. From 2013 to 2019, Adellgene Huntington Disease kit from Blackhills Diagnostic Resources was used in the study of the dynamic mutation (CAG)_n_ *HTT* gene (LRG_763t1) (NM_002111.8), located in the locus 4p16.3 by amplifying the region where is the sequence CAG trinucleotide repeat and with fluorescence detection. This technology is based on the amplification of genomic DNA by PCR, followed by a fluorescent analysis of the size of the PCR fragments obtained by a genetic analyser, using ABI 3130xl sequencer and GeneMapper 4.0 software which allows the conversion of said size into the exactly number of CAG repeats using a weight marker, Liz500TM. The accuracy of sizing is +/− 1 repeat in the normal allele size range and +/− 3 repeats in the expanded allele size range (> 39 CAG repeats). The laboratory participates successfully in the EMQN (*The European Molecular Genetics Quality Network*) external quality control program since 2015, which guarantees the veracity of the results issued with this technique. Raw data is available as supplementary material.

Study patients were classified according to the number of CAG repeats they have in the following categories: normal: 6–26 CAGs; intermediate zone alleles: 27–35 CAGs; incomplete penetrance alleles: 36–39 CAGs; complete penetrance alleles (adult presentation): 40–59 CAGs; complete penetrance alleles (youth presentation): > 60 CAGs [16]. More detailed clinical and familiar information was obtained of the electronic clinical history of the patients and their family members to know the transmission of the disease (paternal or maternal).

The incidence of HD was calculated from the number of new HD cases diagnosed per 100,000 inhabitants per year during the period 2007–2019. Binomial confidence intervals (CI) were also calculated. Annual population estimates were obtained through the Information Systems Management Department of HUMS belonging to the Aragonese Health Service.

### Statistical analyses

The frequency distribution of the percentages of each category for each qualitative variable was calculated. The quantitative variables of the study were explored with the Shapiro-Wilk test (goodness of fit adjustment to a normal distribution) and indicators of central tendency (mean or median) and dispersion (standard deviation or percentiles) were calculated.

Differences between groups according to sex, age at referral, genetic testing results and family history were analysed by hypothesis contrast test, with comparison of proportions (χ^2^) and mean comparisons (Student’s t, Mann-Whitney U, Kruskal-Wallis test) as appropriate. Data were analysed using the Jamovi statistical software and statistical significance was determined at *p* ≤ 0.05 (two-tailed).

## Results

A total of 50 (27.9%) incident cases of HD (CAG repeat length ≥ 36) were identified by genetic testing in the Clinical Genetics Section of the HUMS between January 2007 and December 2019 from a total of 179 persons studied. The remaining 129 of the 179 (72.1%) were HD negative (CAG repeat length < 36). Samples included in this database were sent from 19 different primary healthcare centers and hospitals in the Autonomous Community of Aragon. Demographic and clinical characteristics are shown in Table 1.

**Table 1:** Characteristics of cases undergoing genetic test for HD

	CAG repeats ≥36	CAG repeats < 36	Total
Sex			
Males	21 (42.0)	58 (45.0)	79 (44.1)
Females	29 (58.0)	71 (55.0)	100 (55.9)
Age of testing (years)			
< 20	1 (2.0)	4 (3.1)	5 (2.8)
20–39	14 (28.0)	24 (18.6)	38 (21.2)
40–49	6 (12.0)	20 (15.5)	26 (14.5)
50–59	9 (18.0)	25 (19.4)	34 (19.0)
> 60	20 (40.0)	56 (43.4)	76 (42.5)
Clinical manifestations			
Neurological	21 (42.0)	69 (53.5)	90 (50.3)
Psychiatric	4 (8.0)	2 (1.5)	6 (3.3)
Mixed	12 (24.0)	20 (15.5)	32 (17.9)
Asymptomatic	13 (26.0)	38 (29.5)	51 (28.5)
Types of testing			
Diagnostic	37 (74.0)	91 (70.6)	128 (71.5)
Predictive	11 (22.0)	35 (27.1)	46 (25.7)
Prenatal	2 (4.0)	3 (2.3)	5 (2.8)
Sample			
Peripheral blood	48 (96.0)	126 (97.7)	174 (97.2)
Amniotic fluid	1 (2.0)	1 (0.8)	2 (1.1)
Chorionic villi	1 (2.0)	2 (1.5)	3 (1.7)
Zygosity			
Homozygous	0 (0.0)	47 (36.4)	47 (26.3)
Heterozygous	50 (100.0)	82 (63.6)	132 (73.7)

Demographic, clinical characteristics and genetic test results expressed in terms of absolute and percentage relative frequencies: N (%)

According to the data, 29 (58.0%) females and 21 (42.0%) males were confirmed as HD cases. Of these, 42 (84.0%) reported a suspected or genetically confirmed positive family history, in three cases (6.0%) family history was negative and in the remaining five (10.0%) it was impossible to determine the family or sporadic origin of HD. New mutation events were genetically proven in one case, estimating that the minimum new mutation rate for HD in our population is 2.0%.

**Fig. 1:**
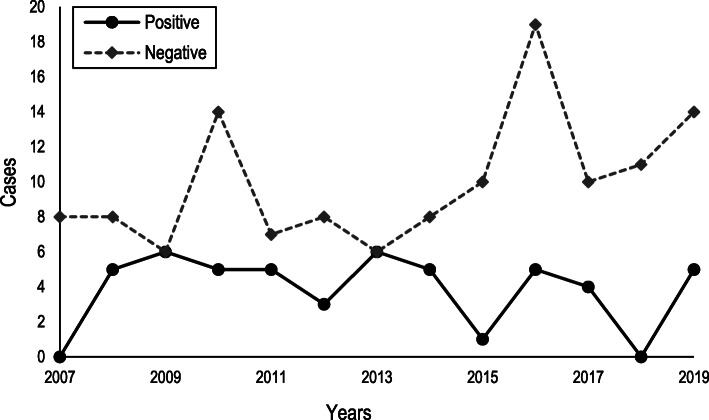
Number of positive and negative genetic test results for HD per year (2007–2019)

Although the change in the diagnostic technique that occurred between the periods 2007–2012 and 2013–2019 has allowed an increase in the number of predictive tests performed (15 (19.5%) *v* 33 (31.7%) predictive test), this fact has not modified significantly the number of positive cases/year (χ^2^ = 19.2; *p* = 0.116). However, it is important to note that 11 (22.0%) of the 50 positive HD cases were identified by performing a predictive test, in the absence of any evident clinical symptom. Number of positive and negative genetic test results for HD per year between 2007 and 2019 is illustrated in Fig. 1. Total population served by the Clinical Genetics Section of the HUMS during the full period was 593,387 person-years on average, resulting in an incidence rate of HD of 0.648 (95% CI 0.587 to 0.709) per 100,000 patient-years, calculated based on the number of newly diagnosed cases by genetic testing. Positive cases/year for females (2.23; 95% CI 1.63 to 2.83) and males (1.62; 95% CI 0.86 to 2.38) were similar, with no statistically significant differences between them (χ^2^ = 0.0761; *p* = 0.783).

The mean age of testing was 52.2 years (SD 18.3; range 19–91 years) and more than 43.0% of all HD cases were diagnosed with more than 59 years. Besides, it was similar in females (mean 50.3; SD 19.0; range 19–90 years) and males (mean 54.7; SD 17.3; range 22–91 years) (*U* = 3894; *p* = 0.700). In addition, age at referral do not show any significant difference over the period of the study (*K* = 20.8; *p* = 0.077). Among cases with negative family history, the mean age at referral was 56.1 (SD 8.1) years, not significantly different from 48.4 (SD 17.0) years on average obtained in cases with positive family history (*U* = 47.5; *p* = 0.495).

The number of CAG repeats in the expanded allele ranged from 37 to 65 with a mean size of 41.8 (SD 5.8) repeats. In 32 (14 paternal and 18 maternal) of the 42 cases with positive family history, the type of inheritance was determined. No significant differences were found between the number of CAG repeats in the expanded allele and the paternal or maternal inheritance (*U* = 114; *p* = 0.648). The minimum number of CAG repeats found in the study population was 9 and the most common CAG length among HD negative individuals was 16.

### Clinical and family history details of patients in whom a prenatal test was performed

The genetic test results for HD, sample type and family history details of the five prenatal tests performed are summarized in Table 2.

**Table 2:** Prenatal test cases

Cases	CAG repeats		CAG repeats of parent at risk for transmitting		Sample type	Family history
	A1	A2	A1	A2		
A	14	38	13	38	CV	HD confirmed mother, aunt and grandfather
B	13	32			CV	Brother of A
C	15	22	22	39	AF	HD confirmed mother and grandfather
D	16	37	18	37	AF	HD confirmed mother and grandfather
E	15	22	22	47	CV	HD confirmed mother, grandfather and great aunt

Distribution of cases by genetic results, sample type and family history in prenatal test performed

*A1* allele, *A2* allele 2, *CV* chorionic villi, *AF* amniotic fluid

Case A and B are both descendants of a 33-year-old asymptomatic female who was a carrier of an incomplete penetrance allele. Fetus A had an incomplete penetrance allele while B had a normal and an intermediate allele. None of these pregnancies were interrupted. Case C and D were prenatal tests performed on a 33 asymptomatic and 30-year-old symptomatic pregnant women, carriers of an incomplete penetrance allele. Fetus C was HD negative while D was in the incomplete penetrance range and the pregnancy was interrupted. Finally, case E was a prenatal test performed on a 33-year-old IVF pregnant woman diagnosed with HD at the age of 30 which resulted in two alleles in normal range.

### Clinical and family history of patients with CAG repeats in the intermediate range

Genetic test results for HD and family history details of the seven tests performed are summarized in Table 3. Frequency of IA in our population (7/308 normal chromosomes) was 2.3%. Despite not being described the relationship between the presence of symptoms in individuals with alleles in the intermediate range, 42.3% of the patients with IAs were genetically studied due to their clinical context with different symptoms of HD.

**Table 3:** Cases with CAG repeats in the intermediate range (27–35) and their relatives with other CAG repeats

Patient	Sex	CAG repeats		Family history	Clinical manifestations
		A1	A2		
1	Male	17	29	HD confirmed two siblings	–
Relatives	Male	19	44	Brother	Depression
	Female	17	44	Sister	Essential tremor, bradykinesia, stiffness in the upper right limb
	Female	17	19	Sister	–
2^a^	Male	16	43	Negative	Generalized dystonia, multiple motor tics
Relatives	Male	19	33	Father	–
	Female	17	33	Sister	–
	Male	17	33	Brother	–
3	Female	18	27	Negative	Generalized choreic movements, muscle contractions
4	Female	17	31	HD suspected father and paternal aunts	Depression, anxiety, phobia, chronic stress
5	Male	18	34	Negative	Progressive instability, choreic movements and restlessness

Distribution of cases by genetic results of intermediate allele and family history in predictive and diagnostic test performed. *A1* allele, *A2* allele 2. ^a^In this case, the proband (patient 2) presented an allele in the full penetrance range, being his relatives the carriers of the intermediate allele

### Clinical and family history details of patients with de novo mutations

In Patient 2, as described in Table 3, a true new mutation event was highly suspected by the identification of an intermediate allele in three asymptomatic first-degree relatives. In this case, expansion of an intermediate allele, paternally inherited, was demonstrated confirming a new mutation event.

There were two symptomatic women without family history of HD that could be considered possible de novo mutations, as additional DNA molecular analysis could not be carried out in their relatives.

## Discussion

More than 43.0% of all HD cases were diagnosed with more than 59 years, being the mean age of testing 52.2 years-old, which confirms the late onset of the HD [2, 16], with no differences between men and women.

Based on the total population served by the Clinical Genetics Section of the HUMS the overall incidence was 0.648 per 100,000 patient-years, no differences were found between men and women. In addition, taking into account the different study periods, similar values were obtained (0.668 in 2007–2012 and 0.631 in 2013–2019).

A few years earlier, in the Basque country and Navarre in Spain (geographical areas adjacent to our community), Ramos-Arroyo et al. determined an incidence of 0.48 and 0.46 per 100,000 patient-years respectively [14], being lower than the incidence found in our population.

In other European countries, the incidence rates calculated were the following, all of them per 100,000 person-years: 0.3 in the province of Ferrara, northern Italy (1990–2009) [17]; 1.8 in Germany (2015–2016) [18]; 0.14 in Iceland (1988–2007) [19]; the minimum incidence rate was 0.22, being able to reach 0.44 in Greece (1995–2008) [20]; 0.25 in Cyprus (1984–2015) [21]; and 0.72 in United Kingdom (1990–2010) [22]. So, the incidence of HD varies from 0.14 to 1.8 per 100,000 person-years in Europe.

In the population of the United States from 2003 to 2016 the incidence rate was 1.22 per 100,000 person-years [23] while in Asian countries it is much lower (0.06 in Korea during 2009–2013 [24] and 0.1 in Taiwan during 2000–2007 [25]). In their review, Pringsheim et al. found a mean incidence of 0.4 per 100,000 person-years. Studies including non-Asian population has a higher incidence (0.1–0.8) than studies with Asian descent populations (0.05–0.1) [26]. No incidence studies have been found in the African population [27], although the prevalence of HD in sub-Saharan areas and southern Africa has been found to be lower than in populations of Caucasian origin [27–29].

The incidence in Aragon is higher than the reported for other Caucasian populations, although exists an apparent heterogeneity that varies both time and place. Indeed, this heterogeneity appeared in the estimations of the HD prevalence too, being lower in studies carried out in Asia and Africa than in those that include predominantly populations of European descent [22, 27, 29]. Differences in diagnostic criteria and case ascertainment could explain this variety. Also, if pre-symptomatic cases were included, to the proportion of individuals opting for a predictive testing, which would increase the number of cases. Since the early 1990s for the HD mutation exists a molecular genetic test for its diagnostic. This way, depending on its availability in the different geographical regions, the estimate of new cases may vary. Finally, some authors have suggested the presence of modifiers of the expression of the disease (genetic or environmental) that could play an important role in the expansions of the HD allele around the world, what would make the population more or less prone to the disease [30, 31].

Given that the genetic mutation presumably originated in north-western Europe [26], it suggests that there could be a founder effect in Aragon. This would be possible since the incidence is higher in this region compared to other European populations, but more similar to the Basque Country and Navarra, neighbouring communities also located in north-eastern Spain. However, it would be necessary to carry out more extensive studies for its confirmation.

This complex landscape contributes to an uncertain epidemiology for HD and incidence rates are probably underestimated despite the progress in the knowledge of this disease in recent years [17].

The most common CAG length in our population among the normal alleles was 16, slightly lower than the length found in European population with 17 CAGs repeats [7]. In Danish population, this size is the most frequent too [32], as well as for Canadians [33].

The mean size of 17.2 (SD 2.8) repeats for Aragonese people was similar to that found in European population, being larger compared to the mean size in Asian countries such as China, Japan and Thailand (16.4, 16.6 and 16.5 repeats respectively) [24]. In Latin America the mean varies from 15 to 17.8 repeats, similar to those reported among Caucasians an Asians [34], being 16 the most common repeat among HD negative individuals in Cuba and 17 in Venezuela [35].

These variations in the normal range of CAG repeats according to the population studied (greater for countries whose offspring are of Caucasian origin than in those of Asian origin) correspond to those observed in the incidence and prevalence of the HD, since both are directly related [34]. In populations with high prevalence of HD is higher the number of CAG repeats in normal chromosomes [35].

Planning a family and prevention of passing on an expanded CAG-repeat to future offspring are the main reasons to apply for prenatal or preimplantation genetic diagnosis [36]. In our population, four prenatal and one preimplantation tests were carried out for 4 mothers affected of HD. In these cases, the importance of genetic counselling was revealed, being necessary the translation of the advances in genetic and in-utero techniques into genetic counselling, taking into account the ethical and scientific principles allowing families to make decisions, including the interruption of pregnancy [37], as one of our cases. It is important because younger generations are likely to be more seriously affected by inheriting a CAG repeat amplification, so the detection of the disease and prenatal tests would make it possible to inform and counsel other family members [14].

The frequency of IA in our population was 2.3%, similar to other general populations being observed in 1–7%, which can be considered commonly present [36]. The 42.3% of cases in this range presented clinical symptoms. Some years ago, IAs were not considered associated with symptomatic HD. Nevertheless, nowadays there is evidence that some individuals with IAs express the disease phenotype, developing HD-like clinical and neuropathological manifestations, as in our study, being more notable in older patients or in those with greater repeats within the range 27–35 [8].

When IA has more than 30 repeats, the probability that an expanded allele could be transmitted to the offspring is higher, being able to reach full penetrance [7]. In addition to the size of CAG, the likelihood of expansion to the disease associated range is highly influenced by the sex of the transmitting parent, being the majority of new mutations of paternal transmission [38], although also could be inherit by maternal line [9]. This explains our only confirmed case of a new mutation in which the father with 33 CAG repeats has been responsible for the transmission of the disease to his son (Patient 2) in the range of complete penetration.

This way during meiosis, IA instability results in de novo expansions, an uncommon but well-known source of new HD cases in individuals with no family history. The minimum new mutation rate for HD in our population was 2.0%, below the new mutation rate for the Basque country and Navarre with a minimum of 4.7% and a potential rate of 8.1% [14], although the new mutation rate for HD is estimated to be 10.0% [39]. In these patients, the negative family history of HD observed could be explained by new mutations, but also it could be due to misdiagnosis in family members, non-penetrance or non-paternity [20]. So further studies will be needed to confirm these findings, including genetic testing of family members even those asymptomatic ones. This fact is important information to take into account when genetic counselling a family with a true de novo HD case [40].

The main limitations of the study were found in the calculation of the de novo rate of new mutations, since in two patients their relatives could not be genetically tested. In addition, the association of clinical symptoms in those patients with IA is controversial. Other diseases with the same type of symptoms should be studied in order to be able to firmly affirm that this symptomatology is due to the presence of these alleles. Finally, the incidence has been calculated with reference to the population that belongs to our health sector, but it may be neglecting patients who, even belonging to this area, have carried out the study elsewhere.

## Conclusions

Despite being a rare disease, the incidence of HD in our population can be considered relevant. The implementation of new techniques in the Genetics Laboratory such as fluorescent PCR has allowed us to determine the exact number of CAG repeats, which is especially important in those patients with triplet expansions in an HD intermediate and/or incomplete penetrance allele, both in diagnostic, predictive and prenatal tests. In addition, this technology enables us to have truthful and reliable results in 12–24 h offering an adequate and timely genetic counselling to the patient and their relatives.

## Supplementary Information

**Additional file 1:** **Suplementary table 1.** Datasets generated and analysed.

## Data Availability

The datasets generated and analysed during the current study are available as Supplementary Material. The website https://www.ncbi.nlm.nih.gov/nuccore/NM_002111 was consulted to obtain reference information about the *HTT* gene.

## Abbreviations

CAG: Cytosine-Adenine-Guanine
CEICA: Comité de Ética de la Investigación de la Comunidad de Aragón
CI: Confidence interval
EMQN: The European Molecular Genetics Quality Network
HD: Huntington disease
HUMS: Hospital Universitario Miguel Servet
IAs: Intermediate alleles
PCR: Polymerase chain reaction
SD: Standard deviation
