# Supplementary material for: Incidence of Huntington disease in a northeastern Spanish region: a 13-year retrospective study at tertiary care centre
Source: BMC Med Genet. 2020 Nov 23;21:233. doi: 10.1186/s12881-020-01174-z (PMC7684714; doi:10.1186/s12881-020-01174-z)
Supplement: Supplementary file 1 — Additional file 1: Suplementary table 1. Datasets generated and analysed. [file 12881_2020_1174_MOESM1_ESM.pdf]

Suplementary table 1. Datasets generated and analysed.

Patient	Date	Sex	Clinical manifestations	Sample	Test	CAGs Allele 1	CAGs Allele 2	Zygosity	CAG Range
1	17/04/2007	F	Neurological	Peripheral blood	Diagnostic	11	16	Het	Normal
2	14/05/2007	M	Neurological	Peripheral blood	Diagnostic	16	16	Hom	Normal
3	07/08/2007	F	Neurological	Peripheral blood	Diagnostic	12	13	Het	Normal
4	13/09/2007	M	Neurological	Peripheral blood	Diagnostic	12	12	Hom	Normal
5	17/09/2007	F	Neurological	Peripheral blood	Diagnostic	12	12	Hom	Normal
6	01/10/2007	M	Neurological	Peripheral blood	Diagnostic	13	12	Het	Normal
7	21/11/2007	F	Neurological	Peripheral blood	Diagnostic	13	18	Het	Normal
8	26/11/2007	F	Neurological	Peripheral blood	Diagnostic	13	13	Hom	Normal
9	22/01/2008	F	Mixed	Peripheral blood	Diagnostic	19	41	Het	Complete penetrance
10	08/04/2008	F	Neurological	Peripheral blood	Diagnostic	11	14	Het	Normal
11	19/05/2008	F	Neurological	Peripheral blood	Diagnostic	9	16	Het	Normal
12	23/05/2008	F	Neurological	Peripheral blood	Diagnostic	16	16	Hom	Normal
13	17/06/2008	M	Neurological	Peripheral blood	Diagnostic	14	14	Hom	Normal
14	19/06/2008	M	Neurological	Peripheral blood	Diagnostic	41	14	Het	Complete penetrance
15	21/08/2008	M	Neurological	Peripheral blood	Diagnostic	11	16	Het	Normal
16	10/09/2008	M	Psychiatric	Peripheral blood	Diagnostic	19	44	Het	Complete penetrance
17	11/09/2008	M	Neurological	Peripheral blood	Diagnostic	13	13	Hom	Normal
18	24/09/2008	F	Neurological	Peripheral blood	Diagnostic	12	16	Het	Normal
19	03/11/2008	M	Neurological	Peripheral blood	Diagnostic	9	9	Hom	Normal

20	11/11/2008	F	Neurological	Peripheral blood	Diagnostic	16	41	Het	Complete penetrance
21	27/11/2008	F	Neurological	Peripheral blood	Diagnostic	11	40	Het	Complete penetrance
22	22/01/2009	F	Neurological	Peripheral blood	Diagnostic	19	36	Het	Incomplete penetrance
23	14/05/2009	M	Neurological	Peripheral blood	Diagnostic	9	40	Het	Complete penetrance
24	18/05/2009	M	Asymptomatic	Peripheral blood	Predictive	13	42	Het	Complete penetrance
25	18/05/2009	M	Asymptomatic	Peripheral blood	Predictive	13	16	Het	Normal
26	22/06/2009	F	Mixed	Peripheral blood	Diagnostic	40	21	Het	Complete penetrance
27	22/06/2009	M	Asymptomatic	Peripheral blood	Predictive	11	11	Hom	Normal
28	22/06/2009	M	Asymptomatic	Peripheral blood	Predictive	11	37	Het	Incomplete penetrance
29	22/06/2009	F	Asymptomatic	Peripheral blood	Predictive	14	11	Het	Normal
30	30/09/2009	M	Asymptomatic	Peripheral blood	Predictive	13	13	Hom	Normal
31	16/10/2009	F	Mixed	Peripheral blood	Diagnostic	14	40	Het	Complete penetrance
32	04/11/2009	M	Asymptomatic	Peripheral blood	Predictive	18	18	Hom	Normal
33	13/11/2009	F	Mixed	Peripheral blood	Diagnostic	14	14	Hom	Normal
34	25/03/2010	M	Asymptomatic	Peripheral blood	Predictive	21	21	Hom	Normal
35	10/05/2010	F	Neurological	Peripheral blood	Diagnostic	13	13	Hom	Normal
36	17/05/2010	F	Psychiatric	Peripheral blood	Diagnostic	17	17	Hom	Normal
37	26/05/2010	F	Neurological	Peripheral blood	Diagnostic	14	26	Het	Normal
38	03/06/2010	M	Neurological	Peripheral blood	Diagnostic	15	21	Het	Normal
39	02/07/2010	M	Mixed	Peripheral blood	Diagnostic	14	14	Hom	Normal
40	06/07/2010	M	Neurological	Peripheral blood	Diagnostic	14	41	Het	Complete penetrance
41	08/09/2010	M	Asymptomatic	Peripheral blood	Predictive	16	19	Het	Normal

42	22/09/2010	F	Psychiatric	Peripheral blood	Diagnostic	17	44	Het	Complete penetrance
43	22/09/2010	M	Mixed	Peripheral blood	Diagnostic	14	41	Het	Complete penetrance
44	24/09/2010	F	Neurological	Peripheral blood	Diagnostic	18	18	Hom	Normal
45	04/10/2010	F	Mixed	Peripheral blood	Diagnostic	14	14	Hom	Normal
46	07/10/2010	F	Mixed	Peripheral blood	Diagnostic	16	16	Hom	Normal
47	13/10/2010	F	Neurological	Peripheral blood	Diagnostic	16	40	Het	Complete penetrance
48	20/10/2010	F	Asymptomatic	Peripheral blood	Predictive	24	41	Het	Complete penetrance
49	08/11/2010	F	Mixed	Peripheral blood	Diagnostic	16	16	Hom	Normal
50	22/11/2010	M	Asymptomatic	Peripheral blood	Predictive	17	29	Het	Intermediate
51	01/12/2010	M	Neurological	Peripheral blood	Diagnostic	18	18	Hom	Normal
52	03/12/2010	M	Neurological	Peripheral blood	Diagnostic	17	17	Hom	Normal
53	17/02/2011	F	Asymptomatic	Peripheral blood	Predictive	13	38	Het	Incomplete penetrance
54	24/03/2011	F	Neurological	Peripheral blood	Diagnostic	15	15	Hom	Normal
55	07/04/2011	F	Asymptomatic	Peripheral blood	Predictive	17	17	Hom	Normal
56	07/04/2011	F	Neurological	Peripheral blood	Diagnostic	19	42	Het	Complete penetrance
57	27/04/2011	F	Asymptomatic	Peripheral blood	Predictive	17	19	Het	Normal
58	29/04/2011	M	Mixed	Peripheral blood	Diagnostic	15	20	Het	Normal
59	09/05/2011	F	Asymptomatic	Chorionic villi	Prenatal	14	38	Het	Incomplete penetrance
60	18/05/2011	M	Neurological	Peripheral blood	Diagnostic	16	16	Hom	Normal
61	19/05/2011	F	Neurological	Peripheral blood	Diagnostic	20	42	Het	Complete penetrance
62	02/09/2011	F	Neurological	Peripheral blood	Diagnostic	13	13	Hom	Normal
63	28/11/2011	M	Neurological	Peripheral blood	Diagnostic	18	18	Hom	Normal

64	14/12/2011	M	Mixed	Peripheral blood	Diagnostic	14	40	Het	Complete penetrance
65	18/01/2012	M	Neurological	Peripheral blood	Diagnostic	13	19	Het	Normal
66	28/02/2012	F	Mixed	Peripheral blood	Diagnostic	13	13	Hom	Normal
67	29/03/2012	M	Neurological	Peripheral blood	Diagnostic	14	14	Hom	Normal
68	23/05/2012	M	Asymptomatic	Peripheral blood	Predictive	18	18	Hom	Normal
69	01/06/2012	F	Neurological	Peripheral blood	Diagnostic	20	20	Hom	Normal
70	15/06/2012	M	Neurological	Peripheral blood	Diagnostic	14	19	Het	Normal
71	13/09/2012	F	Mixed	Peripheral blood	Diagnostic	19	41	Het	Complete penetrance
72	08/10/2012	M	Neurological	Peripheral blood	Diagnostic	14	14	Hom	Normal
73	12/11/2012	F	Neurological	Peripheral blood	Diagnostic	16	22	Het	Normal
74	15/11/2012	F	Mixed	Peripheral blood	Diagnostic	14	42	Het	Complete penetrance
75	20/12/2012	F	Psychiatric	Peripheral blood	Diagnostic	14	40	Het	Complete penetrance
76	14/01/2013	F	Neurological	Peripheral blood	Diagnostic	15	45	Het	Complete penetrance
77	21/01/2013	F	Mixed	Peripheral blood	Diagnostic	18	44	Het	Complete penetrance
78	22/01/2013	M	Mixed	Peripheral blood	Diagnostic	23	23	Hom	Normal
79	13/02/2013	M	Neurological	Peripheral blood	Diagnostic	14	23	Het	Normal
80	19/03/2013	M	Neurological	Peripheral blood	Diagnostic	17	16	Het	Normal
81	11/04/2013	M	Neurological	Peripheral blood	Diagnostic	37	15	Het	Incomplete penetrance
82	30/04/2013	M	Mixed	Peripheral blood	Diagnostic	18	17	Het	Normal
83	30/05/2013	F	Asymptomatic	Peripheral blood	Predictive	42	19	Het	Complete penetrance
84	31/07/2013	F	Neurological	Peripheral blood	Diagnostic	25	17	Het	Normal
85	08/10/2013	F	Neurological	Peripheral blood	Diagnostic	42	17	Het	Complete penetrance

86	10/10/2013	M	Mixed	Peripheral blood	Diagnostic	23	17	Het	Normal
87	25/11/2013	M	Neurological	Peripheral blood	Diagnostic	65	19	Het	Complete penetrance
88	07/01/2014	F	Asymptomatic	Peripheral blood	Predictive	43	17	Het	Complete penetrance
89	07/01/2014	F	Asymptomatic	Peripheral blood	Predictive	20	18	Het	Normal
90	06/02/2014	F	Mixed	Peripheral blood	Diagnostic	42	14	Het	Complete penetrance
91	13/02/2014	M	Neurological	Peripheral blood	Diagnostic	18	17	Het	Normal
92	14/02/2014	M	Psychiatric	Peripheral blood	Diagnostic	23	17	Het	Normal
93	31/03/2014	F	Psychiatric	Peripheral blood	Diagnostic	47	22	Het	Complete penetrance
94	16/04/2014	M	Mixed	Peripheral blood	Diagnostic	40	20	Het	Complete penetrance
95	11/06/2014	M	Mixed	Peripheral blood	Diagnostic	14	13	Het	Normal
96	22/07/2014	F	Mixed	Peripheral blood	Diagnostic	15	17	Het	Normal
97	31/07/2014	F	Asymptomatic	Chorionic villi	Prenatal	32	13	Het	Intermediate
98	28/10/2014	M	Neurological	Peripheral blood	Diagnostic	38	13	Het	Incomplete penetrance
99	06/11/2014	F	Mixed	Peripheral blood	Diagnostic	19	11	Het	Normal
100	19/11/2014	M	Neurological	Peripheral blood	Diagnostic	19	16	Het	Normal
101	10/02/2015	F	Asymptomatic	Peripheral blood	Predictive	17	17	Hom	Normal
102	12/03/2015	F	Neurological	Peripheral blood	Diagnostic	17	15	Het	Normal
103	16/03/2015	F	Mixed	Peripheral blood	Diagnostic	18	17	Het	Normal
104	25/03/2015	M	Mixed	Peripheral blood	Diagnostic	17	17	Hom	Normal
105	07/04/2015	F	Neurological	Peripheral blood	Diagnostic	18	15	Het	Normal
106	14/05/2015	M	Asymptomatic	Peripheral blood	Predictive	19	16	Het	Normal
107	03/09/2015	F	Asymptomatic	Peripheral blood	Predictive	24	16	Het	Normal

108	10/09/2015	M	Neurological	Peripheral blood	Diagnostic	11	11	Hom	Normal
109	20/10/2015	M	Mixed	Peripheral blood	Diagnostic	18	16	Het	Normal
110	05/11/2015	F	Neurological	Peripheral blood	Diagnostic	37	18	Het	Incomplete penetrance
111	09/11/2015	M	Mixed	Peripheral blood	Diagnostic	23	16	Het	Normal
112	19/01/2016	F	Asymptomatic	Peripheral blood	Predictive	17	15	Het	Normal
113	19/01/2016	F	Neurological	Peripheral blood	Diagnostic	26	19	Het	Normal
114	21/01/2016	M	Neurological	Peripheral blood	Diagnostic	43	16	Het	Complete penetrance
115	23/02/2016	M	Asymptomatic	Peripheral blood	Predictive	17	16	Het	Normal
116	23/02/2016	F	Asymptomatic	Peripheral blood	Predictive	33	19	Het	Intermediate
117	29/02/2016	F	Asymptomatic	Peripheral blood	Predictive	39	22	Het	Incomplete penetrance
118	16/03/2016	F	Neurological	Peripheral blood	Diagnostic	27	18	Het	Intermediate
119	07/06/2016	M	Neurological	Peripheral blood	Diagnostic	17	16	Het	Normal
120	08/06/2016	F	Asymptomatic	Peripheral blood	Predictive	33	17	Het	Intermediate
121	08/06/2016	M	Asymptomatic	Peripheral blood	Predictive	33	17	Het	Intermediate
122	20/06/2016	F	Asymptomatic	Peripheral blood	Predictive	24	17	Het	Normal
123	05/07/2016	F	Asymptomatic	Amniotic fluid	Prenatal	22	15	Het	Normal
124	11/07/2016	F	Asymptomatic	Peripheral blood	Predictive	18	18	Hom	Normal
125	22/07/2016	M	Asymptomatic	Peripheral blood	Predictive	22	15	Het	Normal
126	24/08/2016	F	Neurological	Peripheral blood	Diagnostic	16	16	Hom	Normal
127	30/08/2016	M	Asymptomatic	Peripheral blood	Predictive	19	16	Het	Normal
128	31/08/2016	M	Asymptomatic	Peripheral blood	Predictive	16	15	Het	Normal
129	27/09/2016	F	Neurological	Peripheral blood	Diagnostic	23	16	Het	Normal

130	05/10/2016	M	Neurological	Peripheral blood	Diagnostic	40	16	Het	Complete penetrance
131	06/10/2016	F	Asymptomatic	Peripheral blood	Predictive	21	16	Het	Normal
132	31/10/2016	M	Neurological	Peripheral blood	Diagnostic	39	19	Het	Incomplete penetrance
133	31/10/2016	F	Asymptomatic	Peripheral blood	Predictive	39	19	Het	Incomplete penetrance
134	14/11/2016	M	Neurological	Peripheral blood	Diagnostic	16	16	Hom	Normal
135	14/12/2016	F	Neurological	Peripheral blood	Diagnostic	17	17	Hom	Normal
136	04/01/2017	F	Asymptomatic	Amniotic fluid	Prenatal	37	16	Het	Incomplete penetrance
137	19/01/2017	F	Asymptomatic	Peripheral blood	Predictive	21	15	Het	Normal
138	23/02/2017	F	Asymptomatic	Chorionic villi	Prenatal	22	15	Het	Normal
139	27/02/2017	F	Asymptomatic	Peripheral blood	Predictive	16	16	Hom	Normal
140	07/03/2017	M	Neurological	Peripheral blood	Diagnostic	19	18	Het	Normal
141	15/03/2017	M	Neurological	Peripheral blood	Diagnostic	17	15	Het	Normal
142	20/04/2017	F	Neurological	Peripheral blood	Diagnostic	16	16	Hom	Normal
143	19/06/2017	M	Neurological	Peripheral blood	Diagnostic	23	16	Het	Normal
144	12/07/2017	F	Asymptomatic	Peripheral blood	Predictive	38	18	Het	Incomplete penetrance
145	20/07/2017	F	Asymptomatic	Peripheral blood	Predictive	38	17	Het	Incomplete penetrance
146	22/08/2017	F	Neurological	Peripheral blood	Diagnostic	19	16	Het	Normal
147	19/09/2017	M	Neurological	Peripheral blood	Diagnostic	48	17	Het	Complete penetrance
148	02/10/2017	F	Neurological	Peripheral blood	Diagnostic	21	16	Het	Normal
149	08/11/2017	F	Asymptomatic	Peripheral blood	Predictive	19	15	Het	Normal
150	22/01/2018	F	Mixed	Peripheral blood	Diagnostic	20	20	Hom	Normal
151	26/04/2018	M	Neurological	Peripheral blood	Diagnostic	16	16	Hom	Normal

152	07/06/2018	F	Neurological	Peripheral blood	Diagnostic	17	15	Het	Normal
153	26/06/2018	M	Neurological	Peripheral blood	Diagnostic	18	17	Het	Normal
154	09/07/2018	F	Neurological	Peripheral blood	Diagnostic	17	15	Het	Normal
155	18/07/2018	F	Asymptomatic	Peripheral blood	Predictive	16	16	Hom	Normal
156	17/09/2018	F	Neurological	Peripheral blood	Diagnostic	22	16	Het	Normal
157	24/09/2018	M	Neurological	Peripheral blood	Diagnostic	16	15	Het	Normal
158	25/10/2018	F	Asymptomatic	Peripheral blood	Predictive	16	14	Het	Normal
159	07/11/2018	M	Neurological	Peripheral blood	Diagnostic	16	10	Het	Normal
160	10/12/2018	F	Asymptomatic	Peripheral blood	Predictive	24	16	Het	Normal
161	15/01/2019	F	Neurological	Peripheral blood	Diagnostic	22	18	Het	Normal
162	18/03/2019	M	Neurological	Peripheral blood	Diagnostic	41	18	Het	Complete penetrance
163	26/03/2019	F	Neurological	Peripheral blood	Diagnostic	17	16	Het	Normal
164	25/04/2019	F	Asymptomatic	Peripheral blood	Predictive	23	16	Het	Normal
165	13/05/2019	F	Neurological	Peripheral blood	Diagnostic	16	16	Hom	Normal
166	16/05/2019	F	Asymptomatic	Peripheral blood	Predictive	31	17	Het	Intermediate
167	12/06/2019	F	Asymptomatic	Peripheral blood	Predictive	22	16	Het	Normal
168	17/06/2019	M	Mixed	Peripheral blood	Diagnostic	19	18	Het	Normal
169	24/07/2019	M	Neurological	Peripheral blood	Diagnostic	17	14	Het	Normal
170	05/08/2019	F	Mixed	Peripheral blood	Diagnostic	15	12	Het	Normal
171	05/08/2019	M	Neurological	Peripheral blood	Diagnostic	41	13	Het	Complete penetrance
172	13/08/2019	F	Neurological	Peripheral blood	Diagnostic	19	15	Het	Normal
173	28/08/2019	M	Mixed	Peripheral blood	Diagnostic	45	19	Het	Complete penetrance

174	17/09/2019	M	Mixed	Peripheral blood	Diagnostic	41	16	Het	Complete penetrance
175	10/10/2019	F	Neurological	Peripheral blood	Diagnostic	16	14	Het	Normal
176	14/10/2019	M	Asymptomatic	Peripheral blood	Predictive	42	17	Het	Complete penetrance
177	11/11/2019	F	Neurological	Peripheral blood	Diagnostic	16	16	Hom	Normal
178	13/11/2019	M	Neurological	Peripheral blood	Diagnostic	18	10	Het	Normal
179	27/11/2019	M	Neurological	Peripheral blood	Diagnostic	34	18	Het	Intermediate

Raw data of the HD genetic study of 179 individuals who have been referred during the period January 2007 - December 2019 to the Clinical Genetics Section of the Hospital Universitario Miguel Servet, Zaragoza, Spain. M: male; F: female; Het: heterozygous; Hom: homozygous; CAG: cytosine-adenine-guanine.
